# Sphenoid mucopyocele causing bilateral sixth nerve palsy

**DOI:** 10.1093/jscr/rjab607

**Published:** 2022-01-21

**Authors:** Mairead Kelly, Liisa Chang, Parag Patel, Alexander Weller, Rajeev Advani, Raj Lakhani

## Abstract

Sphenoid mucoceles, although rare, should be considered in patients with headache, visual disorders and eye paralysis. Due to close relationships with the orbit and neuromeningeal structures, early recognition is vital. We report the case of a patient who presented with bilateral abducens nerve palsies. At surgery, she was found to have a mucopyocele; this was drained and she required prolonged intravenous antibiotic therapy due to ongoing symptoms and persistent dural enhancement on imaging. A lesion of sufficient size in the clival area has the potential to cause bilateral abducens nerve palsies, though we believe this is the first time it has been described in relation to a sphenoid mucocele. Imaging plays a crucial role in diagnosis, and prompt surgical intervention is essential to avoid serious and permanent complications. The multi-disciplinary team approach is vital—these cases requiring input from ophthalmology, ear nose and throat, microbiology, radiology, neurology and neurosurgery.

## INTRODUCTION

Sphenoid sinus mucoceles are rare, comprising only 1–2% of all paranasal sinus mucoceles [1]. Given their potential proximity to the dura, pituitary gland, optic nerve, cavernous sinus, internal carotid artery and cranial nerves II–VI, sphenoid mucoceles can cause serious complications. Early recognition and management of these lesions is vital to minimise morbidity [2].

## CASE REPORT

A 65-year-old female complained of double-vision and worsening headaches, radiating from the occiput down the neck. Her daughter had commented on her becoming ‘cross-eyed’. She reported two recent falls as she had been unable to judge the position of a chair. There was no jaw claudication, scalp tenderness, light sensitivity or neck stiffness. There had been no recent weight loss, night sweats or history of tuberculosis.

On examination, she had asymmetric weakness of the lateral rectus muscles bilaterally. Both eyes were white, with clear cornea and vitreous. The maculae were normal with no disc swelling. Visual acuity in both eyes was 6/12 unaided and 6/9 with a pinhole. Pupils were equal and reactive to light and accommodation, and there was no relative afferent pupillary defect.

**Figure 1:**
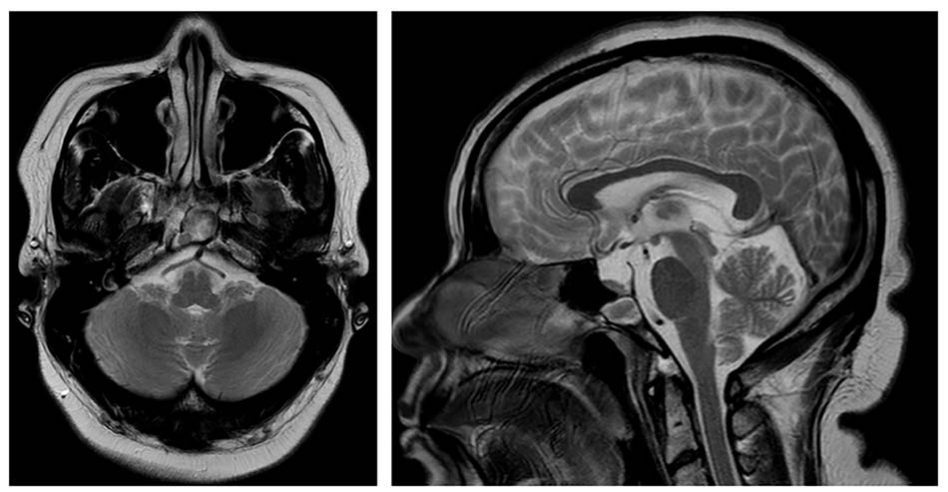
MRI IAMs (Axial T2 weighted image [T2WI] and Sagittal T2WI) showing sphenoid mucocele. The posterior clival cortex is in tact.

The patient was known to have a—previously asymptomatic—sphenoid mucocele. This was detected 6 years prior on a magnetic resonance imaging (MRI) scan of her internal acoustic meatus (IAMs) for dizziness (Fig. 1).

**Figure 2:**
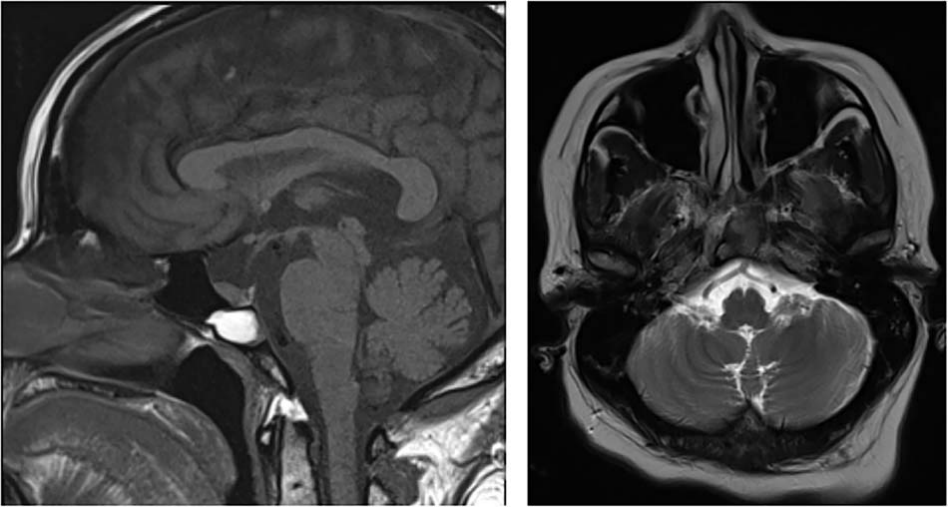
MRI Pituitary (Sagittal T1 weighted image [T1WI] and Axial T2WI) showing the mucocele (high T1WI, low T2WI signal content) and, now, dehiscence at the posterior clival cortex.

**Figure 3:**
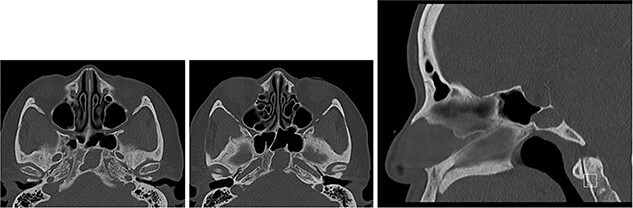
CT of the base of skull (axial images ×2 and sagittal image) showing sphenoid mucocele and paper-thin posterior cortex.

An MRI pituitary undertaken for secondary hypothyroidism 5 years later showed dehiscence at the posterior clival cortex (Fig. 2), and computed tomography (CT) showed the cortex to be paper-thin (Fig. 3).

The patient was referred to an ear, nose and throat (ENT) clinician. She denied any sinonasal symptoms. Follow-up was planned, but had not taken place when she presented with diplopia some 6 months later.

Her past medical history includes glaucoma, Type 2 diabetes mellitus, poorly controlled hypertension and hypercholesterolaemia. She is a non-smoker and drinks alcohol occasionally. Her World Health Organization performance status is 1.

**Figure 4:**
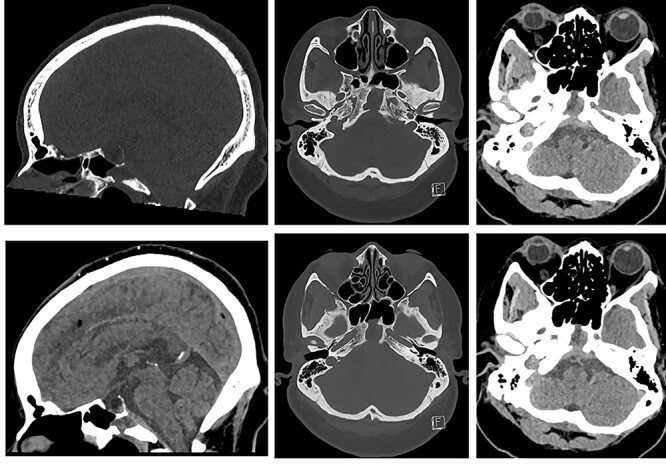
CT of the head (left: sagittal bone and soft tissue windows, and, right: axial bone window and soft tissue window at the same levels ×2) showing sphenoid mucopyocele, retroclival dural thickening and increasing clival dehiscence (anterior and posterior cortex).

A CT head following her acute presentation showed no intracranial event (Fig. 4). There was minimal change in the size of the mucocele. Both anterior and posterior clival dehiscence had increased, reflecting remodelling or pressure deossification. The dehiscence was contiguous with a new smooth plaque of dural retroclival thickening that extended from the petrous ridge/dorsum sellae cranially to the basion caudally, features that are non-specific for extruded proteinaceous mucocele.

Blood tests showed normal white blood cell count (WBC) and C-reactive protein (CRP) and very slightly raised Erythrocyte sedimentation rate (ESR). Nasal endoscopic drainage of the mucocoele was performed. At operation, a thick-walled clival mucopyocoele was encountered inferior to the pituitary fossa. Frank pus was drained. The anterior two-thirds of the mucopyocoele were removed, whereas the posterior wall of the cyst was preserved, as this was adjacent to dehiscent dura. A decision was made not to place a nasoseptal flap as this would risk blocking drainage of the area.

The initial microscopy and culture from the pus sample suggested large amounts of *Staphylococcus aureus* and mixed coliforms. Given the suggestion of dural involvement, intravenous Ceftriaxone 2 g twice daily was started, as well as intravenous metronidazole for anaerobic cover. Subsequent growth showed *Morganella morganii* and *Proteus mirabilis* and the Ceftriaxone was switched to Meropenem. The patient was given bangerter occlusion foil to place on her spectacles to help ease her double-vision, which persisted well beyond surgery.

**Figure 5:**
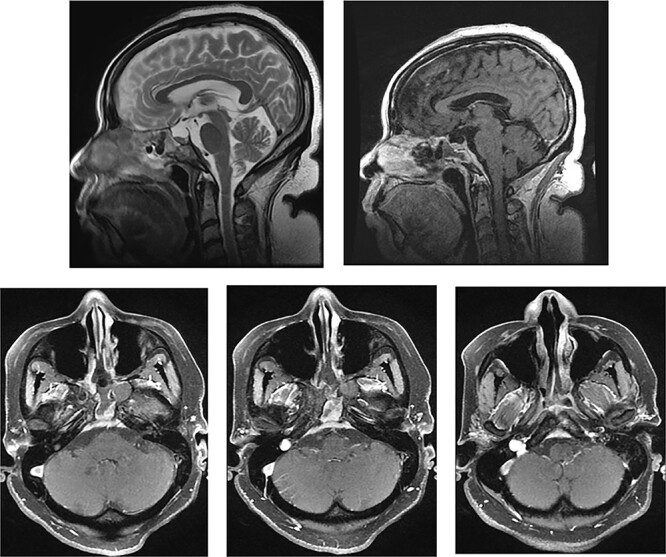
MRI of the head and orbits post-operatively (top row: sagittal T2WI and sagittal T1WI post-contrast; bottom row: axial T1WI post-contrast [from cranial to caudal]) showing fluid-filled sphenoidal sinuses, mucosal thickening and enhancement in the mucocele cavity, as well as persistent retroclival dural thickening.

Due to ongoing symptoms, the patient underwent MRI 11 days post-operatively. This showed fluid-filled sphenoidal sinuses and clival osteomyelitis, extending intracranially to involve retroclival dura (Fig. 5).

**Figure 6:**
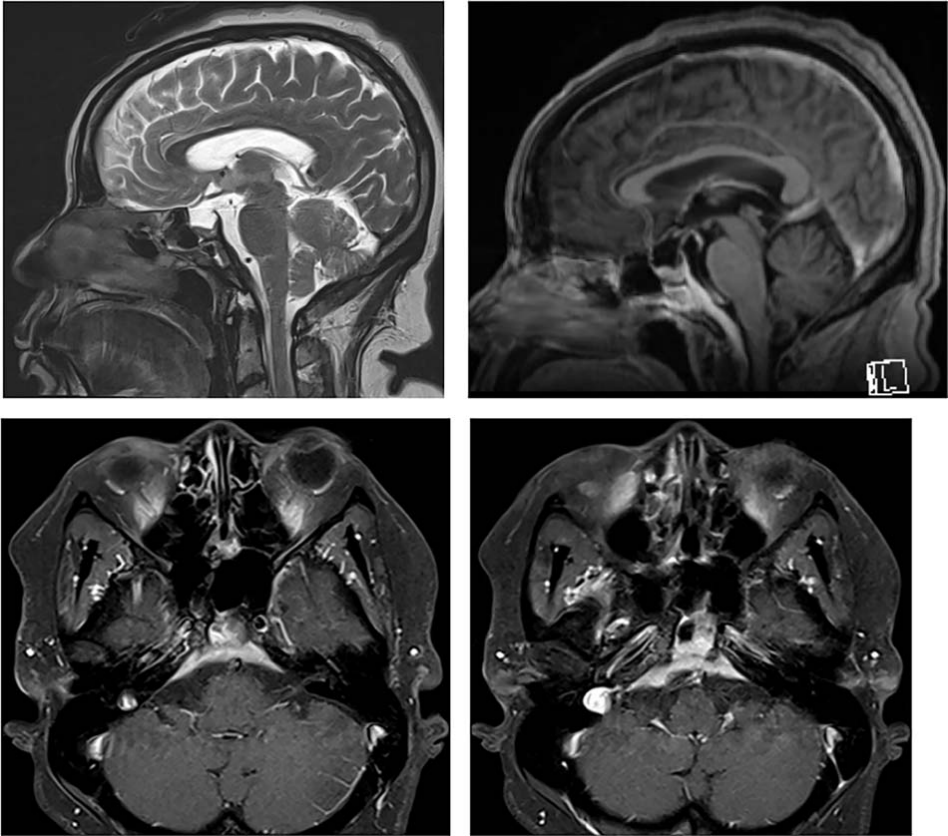
MRI of the head and orbits (top row: sagittal T2WI and sagittal T1WI post-contrast; bottom row: axial T1WI post-contrast at two levels [cranial to caudal]) showing increased retroclival dural thickening; the volume of fluid and mucosal thickening in the sphenoidal sinuses and clival cavity is reduced.

It was therefore decided that prolonged intravenous antibiotic therapy would be needed. She was discharged home with Ceftriaxone intravenously 4 g once daily (for central nervous system cover of *S. aureus*) and Ciprofloxacin orally 750 mg twice daily (to cover *Proteus* and *Morganella*). An MR head after 6 weeks (Fig. 6) showed reduced fluid in the sphenoid sinuses, but slightly increased dural thickening. In view of this, and the patient’s ongoing headache, the antibiotics were continued.

Two months post-operatively, the patient’s headaches had reduced in intensity, her diplopia had resolved, and her abducens nerve palsies were also noted to be much improved.

## DISCUSSION

A literature review showed eight cases of a sphenoid mucocele causing unilateral sixth nerve palsy [3–10]; there was none in which it caused bilateral sixth nerve palsy. In the unilateral cases, all patients also reported headache, and in three, there was concurrent third nerve palsy [6, 9, 10]. Indeed, pupil-sparing oculomotor palsy is the most common nerve palsy associated with sphenoid mucoceles [1].

Given this patient’s history, a sphenoid mucocele was clearly prominent in our differential diagnosis. However, this could also have included any expansive lesions of the sphenoid sinus [5, 9]. The literature shows several reports of bilateral abducens nerve palsy secondary to tumours. These include chordoma, multiple myeloma and diffuse large B cell lymphoma involving the clivus, as well as metastases of Ewing’s sarcoma, small-cell lung carcinoma and lung adenocarcinoma [11].

The anatomical course of the abducens nerves elucidates how a clival lesion of sufficient size can cause bilateral palsies. After piercing the clival dura mater, the abducens nerve enters Dorello’s canal to reach the cavernous sinus. It exits via the superior orbital fissure, to reach the lateral rectus muscle. Given the proximity of the two abducens nerves at the level of Dorello’s canals, a sufficiently large lesion may compress them bilaterally [1, 12], and cause microinfarction, as is likely in this case.

In an asymptomatic patient, it is reasonable to manage a clival mucocele with close observation, but a surgical approach should quickly be adopted when there is evidence of visual impairment or cranial nerve involvement [8]. Prompt surgical management shows the best outcomes [7, 9]. The most common surgical strategy for the management of sphenoid mucoceles presenting acutely is drainage via a nasal endoscopic approach [6–8, 10, 11, 13].

After surgical drainage, antibiotics are appropriate if the lesion is infected. The duration requires close consultation with microbiology and radiology. Patients are likely to need long-term intravenous antibiotics in view of dural enhancement, which can persist for some time following decompression, as in this case [5]. Reports in the literature also show that, as in our patient, abducens palsy can take many months to improve following decompression [5, 6, 9]. Orthoptist input is essential to manage troublesome symptoms during this time.

## CONFLICT OF INTEREST STATEMENT

None declared.
